# Chemical Bonding of AlH_3_ Hydride by Al-*L*_2,3_ Electron Energy-Loss Spectra and First-Principles Calculations

**DOI:** 10.3390/ma5040566

**Published:** 2012-03-30

**Authors:** Kazuyoshi Tatsumi, Shunsuke Muto, Kazutaka Ikeda, Shin-Ichi Orimo

**Affiliations:** 1 Department of Materials, Physics and Energy Engineering, Nagoya University, Chikusa, Nagoya 464-8603, Japan; E-Mail: s-mutoh@nucl.nagoya-u.ac.jp; 2 Institute of Materials Structure Science, High Energy Accelerator Research Organization (KEK), 1-1 Oho, Tsukuba, Ibaraki 305-0801, Japan; E-Mail: kikeda@post.j-parc.jp; 3 Institute for Materials Research, Tohoku University, Sendai 980-8577, Japan; E-Mail: orimo@imr.tohoku.ac.jp

**Keywords:** AlH_3_, chemical bonding, EELS, first principles calculation

## Abstract

In a previous study, we used transmission electron microscopy and electron energy-loss (EEL) spectroscopy to investigate dehydrogenation of AlH_3_ particles. In the present study, we systematically examine differences in the chemical bonding states of Al-containing compounds (including AlH_3_) by comparing their Al-*L*_2,3_ EEL spectra. The spectral chemical shift and the fine peak structure of the spectra were consistent with the degree of covalent bonding of Al. This finding will be useful for future nanoscale analysis of AlH_3_ dehydrogenation toward the cell.

## 1. Introduction

Aluminum trihydride (AlH_3_, alane) has high gravimetric and volumetric hydrogen densities (10 wt % and 149 kg·H_2_/m^3^, respectively). It has been investigated for hydrogen storage applications [1,2,3] after Sandrock *et al*. reported that ball-milling with small amounts of LiH sufficiently accelerated its dehydrogenation kinetics to enable it to be used as an onboard power supply for vehicles [1]. To reveal the mechanism for this accelerated dehydrogenation, it is desirable to analyze the chemical bonding changes of the system on at least sub-micron order.

After the report of Sandrock *et al*., aluminum trihydride has been intensively studied both experimentally and theoretically. In particular, its high pressure phases and phase stabilities have been investigated theoretically [4,5,6] and experimentally [3,6,7]. A theoretical search was conducted for AlH_3_ crystal structures and two crystal structures were proposed: the β and γ-AlH_3_ phases [4]. The crystal structures of the β [8] and γ [9] phases were subsequently analyzed experimentally. The calculated electronic structures (including chemical bonding) of the polymorphs and/or other hydrides have been compared [4,10]. However, apart from reference [11], there have been no experimental studies of their electronic structures. Chemical bonding around H atoms in solid materials cannot be directly investigated by X-ray or electron spectroscopy due to the low scattering powers of H for both x-rays and electrons. In contrast, chemical bonding in compounds in the Al–H–Na system have been theoretically investigated [4]. Alternatively, as has been done only in reference [11], spectroscopic information about the counter element Al in several Al-containing compounds can be systematically investigated to determine the basic chemical bonding of AlH_3_ with the aid of theoretical electronic structure calculations.

We recently investigated dehydrogenation of AlH_3_ by transmission electron microscopy (TEM) and electron energy loss spectroscopy (EELS) [12,13]. We obtained TEM images and electron diffraction patterns during dehydrogenation. Moreover, EELS (including Al core-electron excitation spectra) revealed that single hydride crystals were coated with a thin amorphous alumina layer.

The present study extends this earlier study by investigating the relationship between Al EEL spectra and the chemical bonding of Al in the hydride and other Al-containing compounds. Because the electron energy loss near-edge structure (ELNES) reflects the local electronic structure around the excited atom in the illuminated area, the relationship obtained will provide basic information for future EELS analysis of the change in the local chemical bonding that is responsible for accelerating dehydrogenation.

## 2. Results and Discussion

### 2.1. Al-L_2,3_ EELS

**Figure 1:**
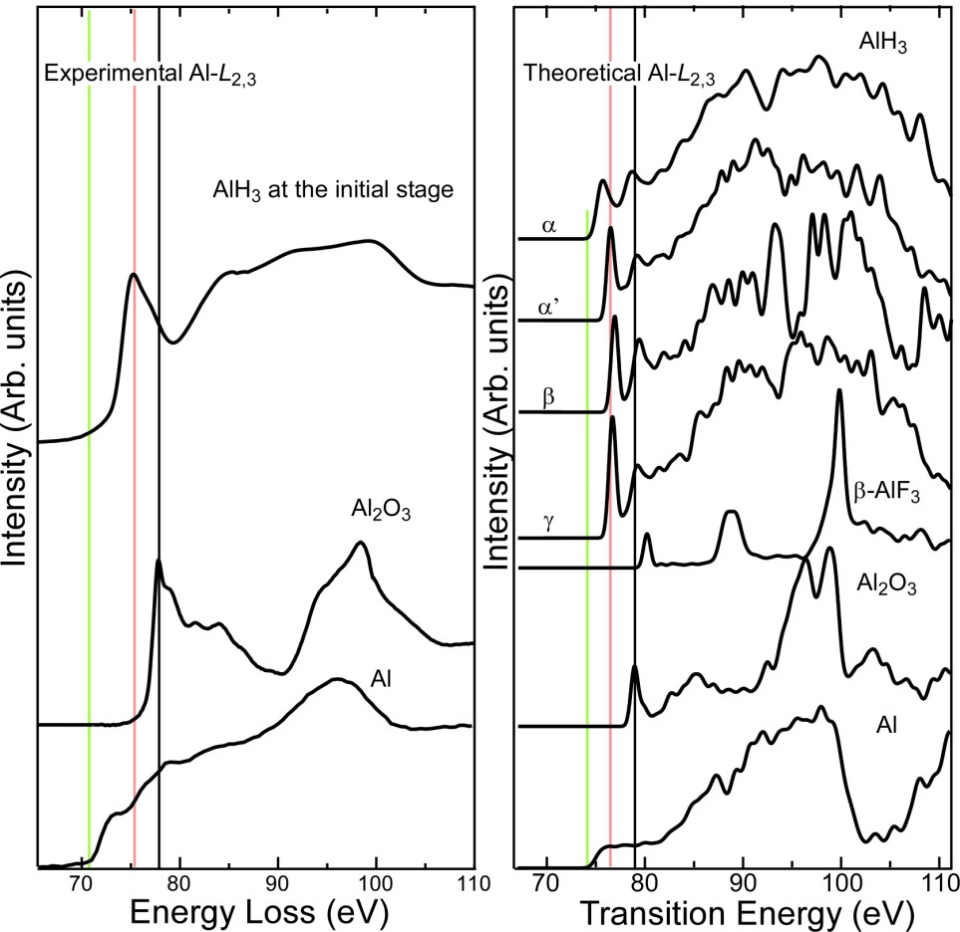
Experimental (**a**) and theoretical (**b**) Al-*L*_2,3_ energy loss near-edge structure (ELNES) of AlH_3_ and reference compounds. The vertical green, red, and black lines indicate the energy positions of the onset of metallic Al spectrum, the first peak of AlH_3_ and the first peak of Al_2_O_3_, respectively.

Figure 1 shows experimental and theoretical Al-*L*_2,3_ spectra of AlH_3_. For reference, it also shows experimental spectra of Al_2_O_3_ and Al to evaluate how well the theoretical calculations reproduce the spectra. Theoretical spectra were calculated for four different AlH_3_ phases (α, α', β, and γ), typical aluminum compounds (Al_2_O_3_ and Al), and β-AlF_3_, which has the same crystal structure as α'-AlH_3_.

The peak profiles, positions, and chemical shifts of the threshold in the experimental and theoretical ELNES of metallic Al and Al_2_O_3_ are in reasonable agreement, although the intensities on the lower energy side (*i.e.*, from the onset of ionization to 10 eV) of the theoretical spectra tend to be smaller than those of the experimental spectra. This underestimation may be a result of excitonic effects beyond the one-electron approximation due to the relatively shallow core-shell excitation [14,15,16]. The characteristics of the experimental spectrum of the hydride are qualitatively consistent with those of the theoretical spectrum. We were unable to identify the phase of the hydride sample from the spectral data because the pre-peak intensities are not very reliable in the theoretical spectra. A higher experimental energy resolution and improved theoretical reproducibility are desirable for phase identification.

To clarify the most characteristic features of the spectral data and the chemical shifts of the Al compounds, the vertical green, red and black lines in Figure 1 respectively indicate the energy positions of the onset for the metallic Al spectrum, and the first peaks in the hydride and Al_2_O_3_ spectra. The calculations correctly reproduce their experimental order. The energy of the first peak in the theoretical β-AlF_3_ spectrum (80.2 eV) is higher than that of Al_2_O_3_ (79.0 eV) by half the energy difference between AlH_3_ and Al_2_O_3_ (2.5 eV).

### 2.2. Electronic Structure near the Band Gap

**Figure 2:**
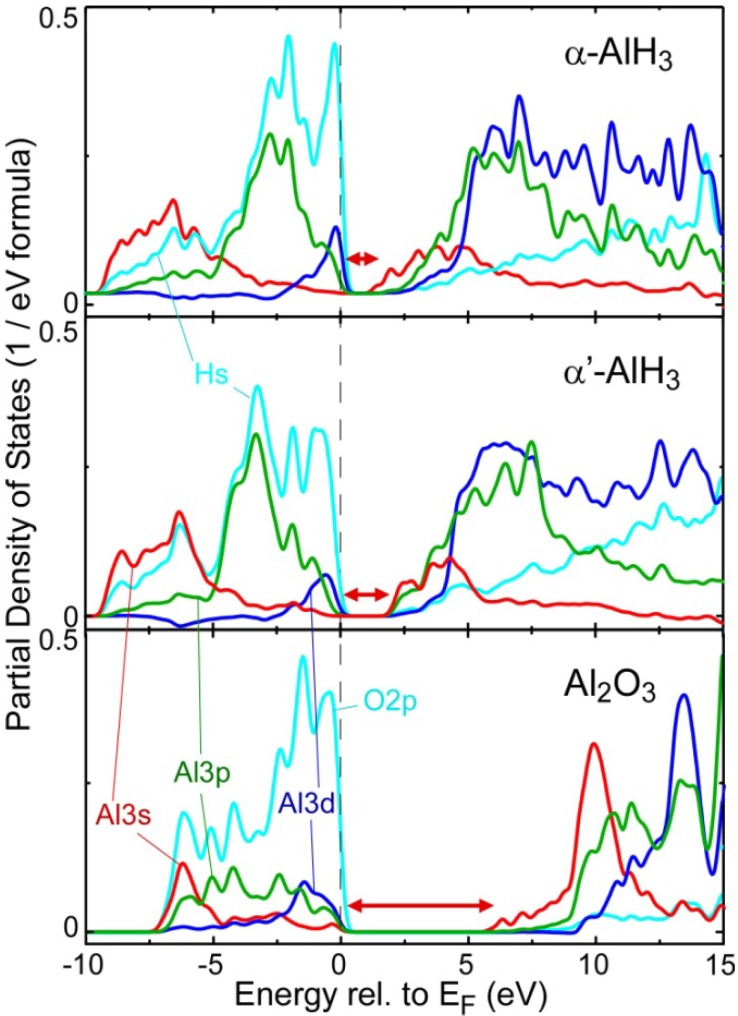
Calculated partial density of states (PDOS) for α-AlH_3_ and Al_2_O_3_. The energy is relative to the Fermi level. The double-headed arrows indicate the band gaps of the electronic structures.

The hydride and other compounds are expected to have different theoretical ground-state electronic structures near the band gap and different chemical bonding: these differences are thought to give rise to the observed chemical shifts in the EELS Al-*L*_2,3_ spectra. Figure 2 shows the partial density of states (PDOS) near the band-gap for α, α'-AlH_3_, and Al_2_O_3_. The band gap widths indicated by the double-headed arrows are respectively 1.4, 2.1, and 5.9 eV for α, α'-AlH_3_, and Al_2_O_3_, indicating that the chemical bonding of AlH_3_ is less ionic than that of Al_2_O_3_. In contrast, the experimentally observed band gap is about 9 eV for Al_2_O_3_. Thus, the present calculation significantly underestimates the band-gap energy, which is common for calculations based on the local density approximation. Improved techniques for calculating the electronic exchange and correlation potential such as the self-interaction-corrected local density approximation [17] are more promising for accurately reproducing the chemical shifts between metallic and insulating compounds; we intend to use such a method in a future study. In AlH_3_ PDOS, the valence bands mainly consist of hydrogen orbitals, indicating that H is anionic. Nevertheless, the contribution of the Al orbitals to the valence bands is greater in AlH_3_ than in Al_2_O_3_. In contrast, the conduction bands are mainly formed by Al orbitals, which are more hybridized with anion (H) orbitals in the hydride than the anion (O) orbitals in the oxide. These compositional differences in the valence and conduction bands confirm that AlH_3_ is less ionic than Al_2_O_3_.

The measured Al-*L*_2,3_ spectra reflect the unoccupied PDOS of Al *s* and *d* symmetry orbitals under the electric dipole approximation. For both AlH_3_ and Al_2_O_3_, the Al 3*s* orbital is dominant over the Al 3*d* orbital at the bottom of the conduction bands. Therefore, the first peaks in the spectra in Figure 1 are roughly assigned to the lower energy states of Al 3*s* bands. The relative energy of the bottom of the conduction bands of α and α’-AlH_3_ with respect to the Al 2*p* inner level is 2.7 (2.1) eV lower than that of the oxide. Since this energy difference is comparable to the experimental chemical shift between these compounds, the difference in the Al ionicity of the theoretical ground electronic structure can well explain the experimental chemical shift. Chemical bonding is discussed in detail in the next section.

The differences between the two hydride polymorphs are rather small. The other hydride phases, which are not shown in Figure 2, had similar PDOS to that of the α' phase. At the conduction band bottom, Al 3*p* is less hybridized with Al 3*s* in the α phase than in the α' and the other hydride phases. This might be responsible for the differences in the pre-peak structures of the polymorphs in the theoretical Al *L*_2,3_ ELNES, although the incorporated excitonic effects should be more rigorously considered.

### 2.3. Covalent Bond Strength

**Figure 3:**
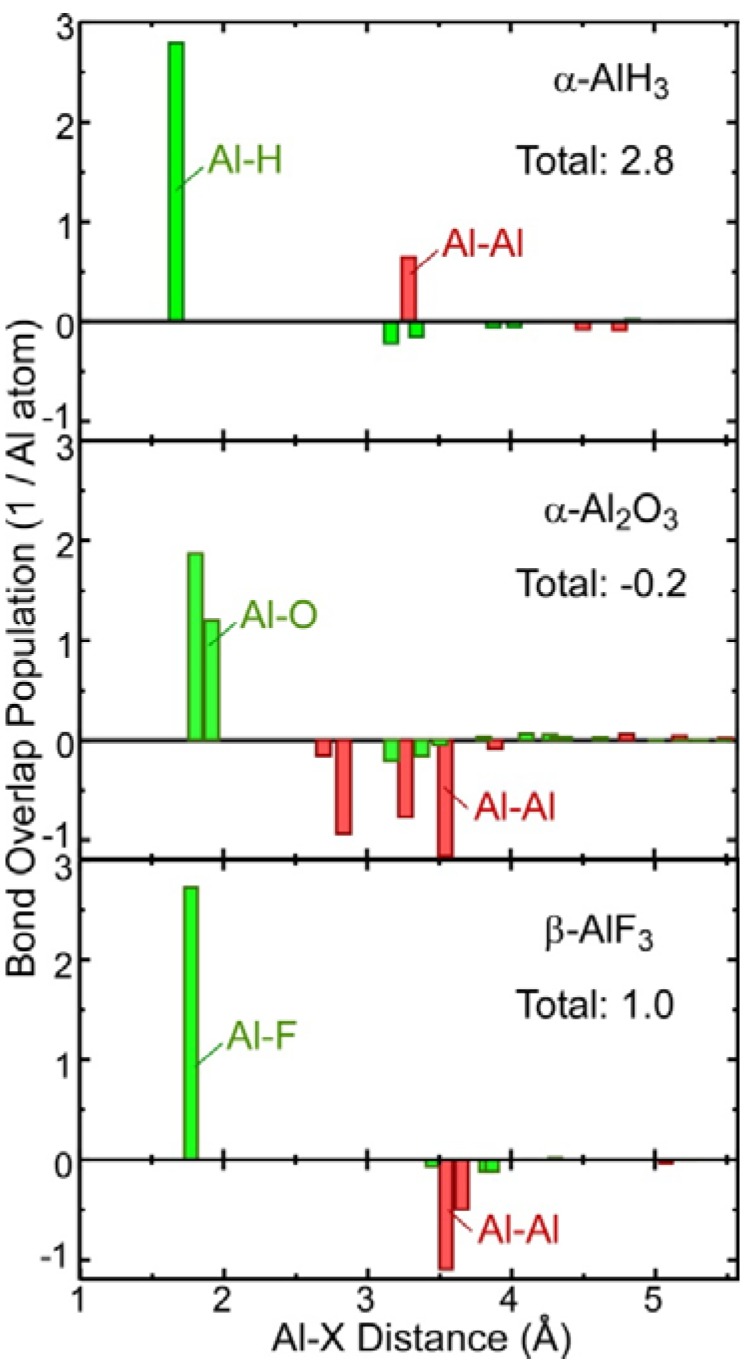
Plots showing the calculated bond overlap population (BOP) and the corresponding atomic distances between an Al atom and its near neighbors. The number in each graph indicates the sum of the BOPs.

To investigate the covalent bond strength between the Al and its neighboring atoms, Figure 3 plots the bond overlap population (BOP) between each atom pair with respect to the interatomic distance. A positive BOP indicates that the covalent bond charge accumulates between the atom pair. On the other hand, a negative BOP indicates that the electron charge between the atoms is deficient relative to that of the superposed atomic electron densities, which is regarded as an antibonding mechanism between the atoms. The hydride contains a significant covalent bond charge for both Al–Al and Al–H. In contrast, Al_2_O_3_ has large negative BOPs for Al–Al, resulting in a negative total BOP of −0.2 per Al atom. This result implies that an alternative mechanism, namely an ionic bonding mechanism, is dominant for Al_2_O_3_. β–AlF_3_ has a similar interatomic distance distribution to that of α-AlH_3_, which is consistent with β–AlF_3_ having an identical crystal structure to α'-AlH_3_. However, unlike for the hydride, all Al–Al pairs have negative BOPs.

### 2.4. Comparison of Chemical Bonding between the Al-Containing Compounds

Table 1 list the theoretical band-gap energy, the Al effective charge, and the sum of BOPs for Al–X (X = Al, H, O, F) to characterize the chemical bonding of the calculated Al compounds. The other hydride phases, which are not shown in Table 1, had similar values to those of the α and α' phases. The Al effective charge represents the number of valence electrons belonging to the Al orbitals. A value smaller than 3 indicates cationic Al. In all of the three rows, the values for the hydrides are intermediate between those of metallic Al and Al_2_O_3_. The ionic bonding or the covalent bond strengths of the hydrides are intermediate between those of Al and Al_2_O_3_.

Except for the smallest BOP of Al_2_O_3_, the listed data indicate stronger ionic bonding toward the right-hand side. The exception may be ascribed to the crystal structure, since the Al–Al distances are much shorter in Al_2_O_3_ than in the hydride and fluoride (see Figure 3). The significant overlap between the Al orbitals and their antibonding interactions results in the lowest total BOP.

The threshold of the Al-*L*_2,3_ edges in Figure 1 is shifted in a manner that is consistent with the overall trend for the chemical bonding in the compounds. In the theoretical spectrum of metallic Al, we can see a number of small peaks located continuously. In contrast, the number of fine peaks in the spectra decreases in the order AlH_3_, Al_2_O_3_, and AlF_3_ with increasing peak intensities and peak distances. Thus, the consistency between the spectra and theoretical data in Table 1 indicates that both the chemical shift and the fine peak structure of the Al-*L*_2,3_ spectra may be experimental indicators for the degree of covalent bonding in the Al-containing compounds. These indicators may help clarify the chemical bonding changes of AlH_3_ in a localized area (for example, changes during the accelerated dehydrogenation).

**Table 1:** Comparison of calculated band gap, effective charge, and BOP of Al-containing compounds (including AlH_3_).

	Al	AlH_3_ (α, α')	Al_2_O_3_	AlF_3_
LDA band gap (eV)	0.0	1.4, 2.1	5.9	7.2
Al effective charge	3.0	2.8, 2.8	2.1	1.8
Sum of BOP Al-X (1/Al atom)	3.6	2.9, 2.7	−0.2	1.0

## 3. Experimental and Theoretical Section

### 3.1. EELS Measurements

The observed hydride sample was prepared by the chemical reaction between LiAlH_4_ and AlCl_3_ in an ether (99.5% purity Et_2_O) solution. Prior to preparing TEM specimens, we confirmed that the powder x-ray diffraction pattern of the sample was that of α-AlH_3_ [3,12]. Further details about the sample preparation and characterization are given in references [12,13].

Electron-irradiation-induced dehydriding of AlH_3_ has been observed [13]. A single crystal of AlH_3_ was decomposed into metallic Al nanoparticles, while its external shape remained almost unchanged. We carefully measured EELS from the hydride by reducing the electron dose to be sufficiently low to avoid its instantaneous decomposition and also to identify the phase by electron diffraction and EELS. Al-*L*_2,3_ ELNES was then isolated after subtracting the pre-edge background by a power law. Since the Al-*L*_2,3_ ELNES of the hydride has a very low signal to noise ratio (<5) due to the low electron dose, the spectrum were processed by the Pixon method [18] to remove statistical noise.

### 3.2. Theoretical Calculations

The theoretical ELNES was calculated by first-principles calculations with a local approximation to the density functional theory so as to investigate the chemical bonding between Al and its surrounding atoms. Prior to the spectral calculations, the crystal parameters of the structures were fully optimized by another first-principles procedure, the projected augmented-wave method [19,20] to reduce the computational cost. For the theoretical ELNES calculation, we adopted the orthogonalized linear combination of atomic orbital (OLCAO) band method [21] because the atomic orbital basis of the method straightforwardly provides the chemical bonding of the structures, as described above. To reasonably account for the core-hole effects [22], an Al 2*p* hole was introduced to a supercell consisting of approximately 100 atoms. The size of the supercell was sufficiently large to neglect the unrealistic interactions between the core holes [23]. The transition probabilities from the Al 2*p* to the unoccupied states were calculated within the electric dipole approximation. These were integrated over the whole Brillouin zone of the supercell using a 2 × 2 × 2 k-point mesh. The transition energy of the theoretical spectrum was obtained from the difference of the total electronic energy of the supercell at the final core hole induced state and that of the ground state. The final spectrum was broadened by convoluting it with a Gaussian function with a full width at half maximum (FWHM) of 0.8 eV. The small energy splitting of 0.07 eV between the Al 2*p*_1/2_ and 2*p*_3/2_ levels was neglected for simplicity.

The chemical bonding differences probed by the spectral difference between AlH_3_ and other Al containing compounds were analyzed based on Mulliken population analysis of atomic orbitals [24]. The PDOS, BOP, and effective charge were calculated by using the overlap integral between atomic orbitals and their coefficients for wavefunctions [25]. This population analysis of the OLCAO method aids intuitive understanding because it uses atomic orbital basis sets, which is one of the main benefits of the OLCAO method. PDOS was constructed by convoluting the orbital population with a Gaussian function (FWHM: 0.4 eV). We chose a smaller FWHM to clearly evaluate the atomic orbital contribution near the band gap.

## 4. Conclusions

We have investigated the chemical bonding of AlH_3_ by means of Al-*L*_2,3_ ELNES and its first-principles band calculation. The results obtained are summarized as follows:

(1) Experimental Al-*L*_2,3_ ELNES exhibited clear chemical shifts between metallic Al, AlH_3_, and Al_2_O_3_. The threshold of the hydride spectrum was intermediate between those of Al and Al_2_O_3_. The first-principles calculation reasonably reproduced the shape and chemical shift of the experimental spectra.
(2) Theoretical chemical bonding data obtained by Mulliken population analysis of the calculated electronic structures revealed that the ionic bonding and covalent bonding strengths of the hydride were intermediate between those of Al and Al_2_O_3_.
(3) The theoretical Al-*L*_2,3_ spectra and chemical bonding between the four Al compounds (Al, AlH_3_, Al_2_O_3_, and AlF_3_) exhibited systematic trends between the chemical bonding and their spectral features (fine peak structure and chemical shift). These trends are expected to be useful for further TEM-EELS nanoscale analysis of the chemical bonding changes that occur during the dehydrogenation of AlH_3_ hydrides.
